# "May I help you?" – Evaluation of the new student service at the reception desk during the clinical courses at the Department of Operative Dentistry and Periodontology as a part of a longitudinal curriculum of social and communicative competences for dental students

**DOI:** 10.3205/zma000973

**Published:** 2015-08-17

**Authors:** Nora Lichtenstein, Isabelle Ensmann, Rainer Haak, Houda Hallal, Jana Kupke, Jan Matthes, Michael Noack, Michael Wicht, Christoph Stosch

**Affiliations:** 1 Universität zu Köln, Medizinische Fakultät, Studiendekanat, Köln, Deutschland; 2 Universität zu Köln, Zentrum für Zahn-, Mund- und Kieferheilkunde, Poliklinik für Zahnerhaltung und Parodontologie, Köln, Deutschland; 3 Universität Leipzig, Poliklinik für Zahnerhaltung und Parodontologie, Leipzig, Deutschland; 4 Universität zu Köln, Institut für Pharmakologie, Köln, Deutschland

**Keywords:** dentistry, longitudinal curriculum, social and communicative competences, teamwork skills

## Abstract

**Objectives: **Since 2009, the University of Cologne has been developing a longitudinal curriculum for teaching social and communicative skills to dental students (LSK-Dent) based on the recommendations of the Association for Dental Education in Europe (ADEE). As a part of this curriculum it was considered to develop a reception service in the undergraduate treatment courses of the Department of Operative Dentistry and Periodontology involving the organizational and administrative handling of the patients by the students. Students should gain an insight into everyday practice and the reception service should function as a learning environment for social und communicative competences. This article introduces the LSK-Dent project, the implementation of the reception service and presents initial evaluation results.

**Methods: **Patients (n=575) and students (n=53) filled out a questionnaire. Additionally, four semi-structured interviews with students were conducted.

**Results: **The reception service was successfully implemented and endorsed by the students. First indications suggest that the reception service was well received by students as a learning environment for social und communicative competences and viewed as an opportunity to gain an insight into everyday practice.

**Conclusion: **The reception service is an innovative addition to the treatment courses and an example for transforming an already existing reality in a course into a new learning environment for students. To what extent the implementation of reflexive elements can increase the subjectively perceived additional benefit by students, has to be addressed in further studies.

## Zusammenfassung

**Zielsetzung: **An der Universität zu Köln wird seit 2009 auf der Basis der Empfehlungen der Association for Dental Education in Europe (ADEE) ein longitudinales Curriculum zur Vermittlung von sozialen und kommunikativen Kompetenzen in der Zahnmedizin (LSK-Dent) entwickelt. Im Rahmen dieses Curriculums wurde überlegt, einen Rezeptionsdienst in den Behandlungskursen der Poliklinik für Zahnerhaltung und Parodontologie zu entwickeln, welcher die organisatorische und administrative Abwicklung der Patienten durch die Studierenden beinhaltet. Durch ihn sollen die Studierenden Einblicke in Praxisabläufe erhalten und er soll als Lernwelt für soziale und kommunikative Kompetenzen fungieren. Dieser Beitrag stellt sowohl das LSK-Dent Projekt als auch die Umsetzung des Rezeptionsdienstes vor und es werden erste Evaluationsergebnisse berichtet.

**Methodik: **Es wurden Patienten (n=575) und Studierende (n=53) mittels Fragebögen befragt. Zusätzlich wurden vier halbstandardisierte Interviews mit Studierenden geführt

**Ergebnisse: **Der Rezeptionsdienst konnte erfolgreich implementiert werden und wird von Patienten und Studierenden gut angenommen. Es zeigen sich erste Hinweise, dass der Rezeptionsdienst von den Studierenden als eine Lernwelt zum Trainieren von sozialen und kommunikativen Fähigkeiten wahrgenommen wird und dass die Studierenden durch ihn einen Einblick in Praxisabläufe erhalten.

**Schlussfolgerung:** Der Rezeptionsdienst stellt eine innovative Ergänzung der bisherigen Behandlungskurse dar und zeigt beispielhaft die Umdeutung bestehender Realitäten in modellierte Erfahrungs- und Lernwelten für Studierende. Inwiefern reflexive Elemente – konzeptionell eingebracht – den subjektiv wahrgenommenen Mehrwert erhöhen können, muss in weiteren Untersuchungen geklärt werden.

## 1. Introduction

### 1.1 Social and communicative competences in dentistry

The positive effects of a good dentist/doctor-patient-relationship are well documented and the training of social and communicative competences during the academic studies of dentistry is currently attracting considerable attention [1], [2]. Especially in dentistry the patient-centered consultation is increasingly regarded as an important step prior to the actual dental treatment. Patients prefer to be included in the decision making [3]. Besides the technical and medical expertise, the success of a dental treatment is distinctively influenced by the dental-patient-relationship [4]. This relationship in turn depends on the quality of the communication between dentist and patient [5].

A lack of social and communicative competences of dentists does not only have an impact on the patient treatment, but also on the job-related satisfaction of the dentists. Especially young professionals feel well trained regarding technical skills, but interpersonal relationships as a part of their everyday practice and aspects that concern the management of a practice are perceived as stressful [6], [7], [8]. 

Based on these results, associations, committees and working groups include social and communicative competences in their recommendations and educational standards: For example the educational standards for dentists published by the Association for Dental Education in Europe (ADEE) [9], the Baseler Consensus-Statement [10] or the Health Professions Core Communication Curriculum (HPCCC) [11]. Thus social and communicative competences are considered as central aspects of the dental profession, they are playing a minor role in the education of dentists [12]. Currently there is no uniform curriculum for social and communicative competences in dental schools in Germany, even though the effectiveness of such curriculums has been documented [e.g. [13]]. In the upcoming “National Competence-based Learning goal Catalogue Dentistry” expected to be passed in 2015 and in the upcoming new licensing regulation for dentists similar recommendations are expected to be included [14]. 

#### 1.2 Teaching of social and communicative competences in the dental education at the University of Cologne

As a part of the program *Innovation and Teaching* (“Innovation und Lehre”) (supported through student tuitions till 2011) the project *Longitudinal Curriculum of Social and Communicative Competences for Dentists* (“Longitudinalcurriculum soziale und kommunikative Kompetenz für Zahnmediziner”) (LSK-Dent) was founded in 2009. The purpose of this project was to develop and implement an evidence-based curriculum addressing the dental-patient communication. Based on the ADEE-criteria (mentioned above) new courses were developed and implemented (see table 1 (Tab. 1)). One aim was to create a link between already existing courses and new learning contents, to allow the new course elements to be integrated in the existing courses without leading to an overload (particularly time commitment) for the students.

Another focus was the sustainable implementation of the new contents. Therefore trainings for lecturers were offered, so that the new course elements can be adopted by as many lecturers as possible.

Within the framework of the established course structure the focus is not only on teaching concrete techniques, as for example participative decision making, but also on facilitating self-reflection as a higher level skill, so that the students can deal with their professional and personal development in a critical manner as mentioned in recommendations for adult education [[15], p.60 ff.]

The new topics are presented in a teaching-learning-spiral with different focuses (see table 1 (Tab. 1)). An additional motivation is created by the fact that certain topics are included in the exams in the sense of “constructive alignments” [[15], p.95 ff.]: communication skills and social competences are tested in form of an OSCE station in the first semester, the sixth semester and in the final exam.

#### 1.3 Development of the idea of a reception service in the undergraduate treatment courses as a integrated part of the LSK-Dent 

Due to newly built facilities of the Centre of for Dental and Oral Medicine at the University of Cologne, in which the clinical treatment courses are held, it was possible to create a separate reception desk for the patients of the treatment courses. Until then the patients had to register themselves at the main reception desk of the policlinic and wait in the main waiting room, until they got picked up and brought to the treatment room by their dental student individually. With the newly available space the question arose whether the students themselves could take care of the new reception desk. The goal of the new “reception service” was to facilitate a quick and direct patient registration, to provide an insight view into everyday practice for the students and to create a learning environment for social and communicative competences, especially to foster inter-professional competences. Studies indicate that the reciprocal understanding of roles of students of different profession, which will be part of a dental team, only partially corresponds [16]. Students, who take over tasks usually conducted by non-dental-employees during the receptions-service, get the chance to experience this role in the dental team and to develop a realistic view of the role and tasks of non-dental team members.

The following questions were derived from that idea:

- Is it feasible that dental students manage organizational and administrative aspects of the patients’ registration process as a part of a reception-service?
- Do students and patients accept the fact that students conduct non-medical work during the treatment courses of the Department of Operative Dentistry and Periodontology?
- Do students perceive the reception-service as a learning environment for social and communicative competences, in particular for teamwork skills?

## 2. Methods

### 2.1 Development and introduction of the reception service in the treatment courses of the Department of Operative Dentistry and Periodontology

Employees of the Administration Department, the Office of Student Affairs of the Medical Faculty, the Department of Operative Dentistry and Periodontology and of the working group LSK-Dent developed a concept for the implementation of the reception service in the treatment courses I and II of the Department of Operative Dentistry and Periodontology during the 7th and 9th semester by the end of 2010 and the beginning of 2011. According to the involved employees the following tasks were considered legitimate to be carried out by the students who are in charge of the reception service: first contact with the patients (scheduling of the appointments and welcoming of the patients), certain aspects of the administrative process (preparing the billing, issuing certificates), answering the phone and discharging patients at the reception. Two students are in charge of the reception desk during the entire treatment course for three days in a row. For further information about the reception service see Attachment 1.

At the beginning of the course the students receive instructions in a two-hour seminar addressing service quality and patient management by an external coach (communication coach, Bertelsmann Academy Gütersloh, with the main focus on sales and service). The students practise in role-plays how to talk to patients on the phone, how to react appropriately to complaints and how to communicate with each other in front of patients. One main focus is on the professional attitude towards co-workers and patients, which enables respectful and deescalating communication. Additionally the students receive an introduction to the procedures at the reception desk and get the chance to practise them under the supervision of a faculty member. During the treatment course lecturers are available for the students, if questions occur. In addition students are encouraged to share their experiences in the form of a handover in form of a verbal exchange of important information between the students who already conducted the reception service and students who are next.

The following potential training area for the reception service were derived from the ADEE-criteria (see table 2 (Tab. 2))

#### 2.2 Evaluation

About 1500 patients were treated in the undergraduate treatment courses during the summer semester 2011 and the winter semester of 2011/2012. All of them received an “answer card” (see Attachment 2) and were asked to fill it out after their treatment. The patients were asked to state if they think the greeting and discharge at the reception desk needs improvement (four item scale: “no improvement necessary” to “major improvements necessary”). They were also asked to rate their overall satisfaction with their appointment in the treatment course. The response rate was 38%. In addition the students attending the treatment course were asked to fill out a questionnaire addressing different aspects of the reception service (likert type scale, see Attachment 3). 53 of the 98 students (54%) filled out the questionnaire. Students and patients had the option to state further comments on the questionnaire. As these comments were unspecific and didn’t refer to the reception service itself, further analysis was omitted. 

In addition semi-standardized interviews were conducted with students to explore which previously derived training areas of the reception service were perceived by the students. Four students (9^th^ semester, 3 female, 1 male), who volunteered, were interviewed. The questions in the interview (see Attachment 4) were derived from the previously mentioned training areas of the reception service derived from the ADEE-criteria (see table 2 (Tab. 2)). The interviewees were German, between 23 and 29 years old and conducted the reception service between three and seven times. None of them had professional work experience as a dentist. The interviews were conducted by a member of the LSK-Dent working group, who is not functioning as a lecturer in the treatment courses, and took 10 minutes on average. The interviews were transcribed literally. The students’ answers were assigned to the previously mentioned training areas (see table 2 (Tab. 2)).

## 3. Results

### 3.1 Service quality and patient satisfaction

**Figure 1:**
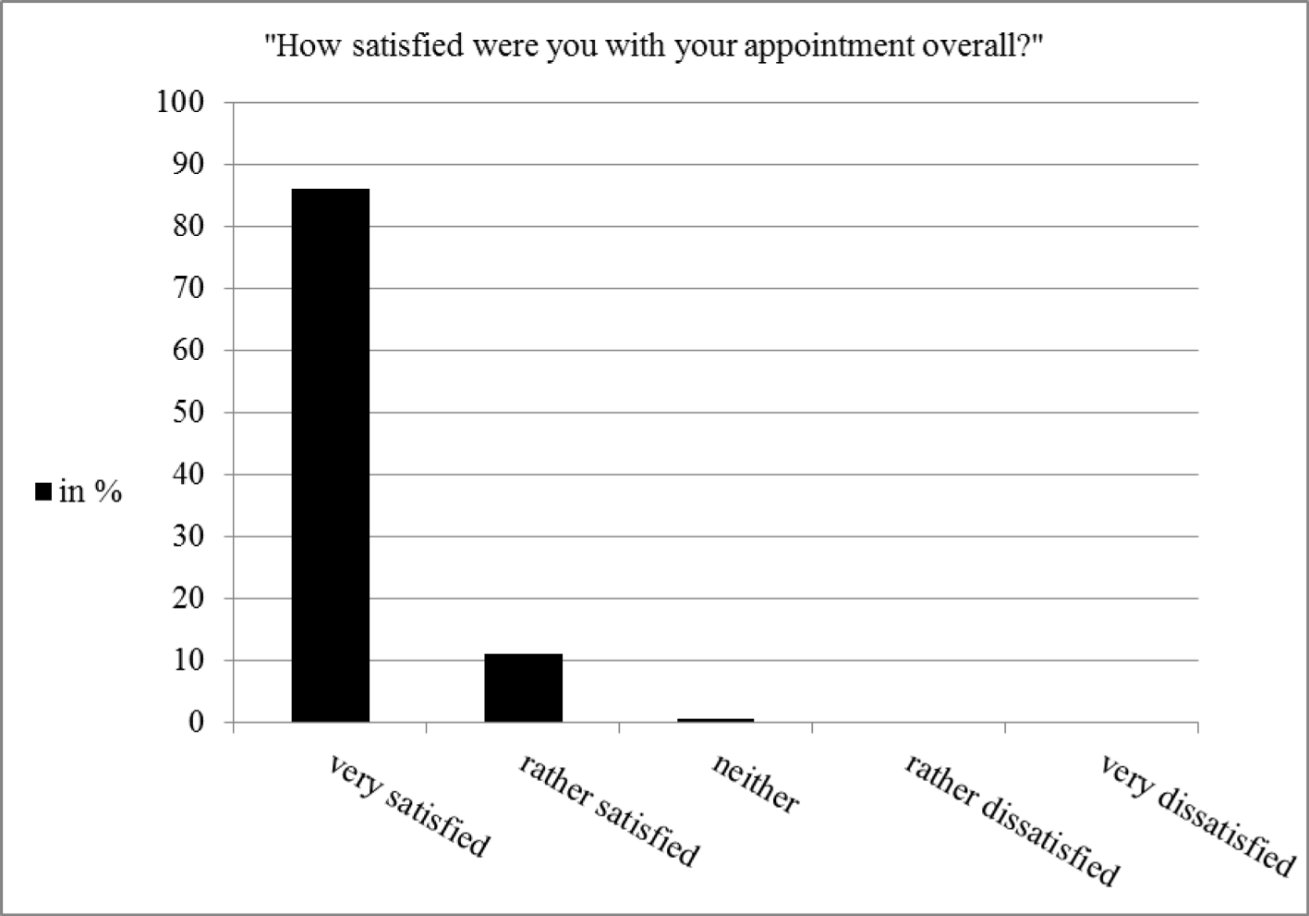
Subjective patient satisfaction with their appointment in the treatment course (n=575).

A great majority of the patients that filled out the answer cards (86%) stated that they were very satisfied with their appointment in the treatment course (see figure 1 (Fig. 1)).

**Figure 2:**
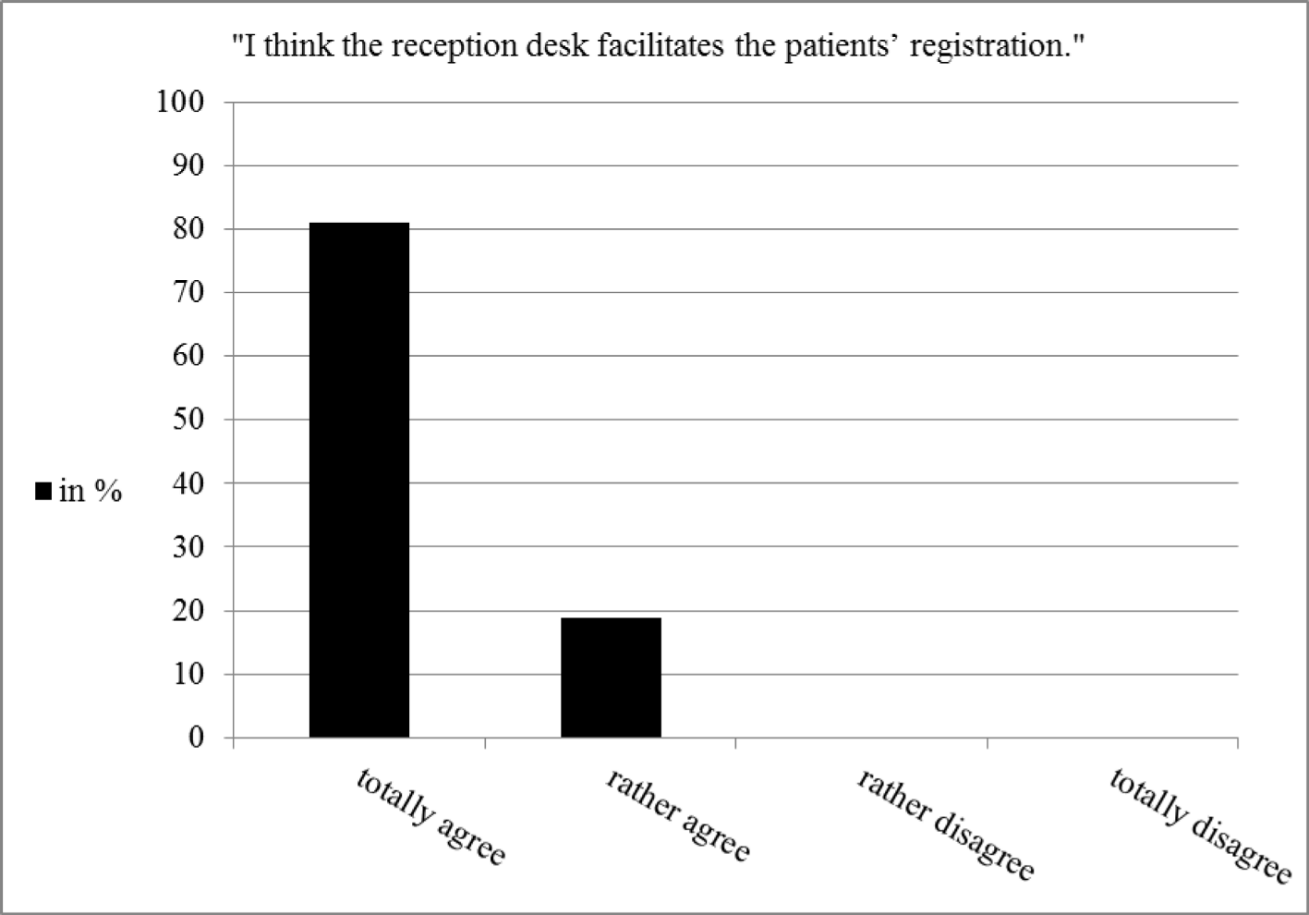
Students‘ assessment of patients registration (n=53)

As reported by the patients, greeting (87%) and discharge (89%) of the patients by the students at the reception desk need no improvement. According to patients’ estimation the majority had to wait no longer than 10 minutes (64%), 24% had to wait up to 20 minutes and 9% more than 20 minutes. 85% thought the waiting time was reasonable, 3% thought it was too long. These results correspond to the students’ statement indicating the new processes lead to an easier registration for the patients (see figure 2 (Fig. 2)).

#### 3.2 Insight into everyday practice

**Figure 3:**
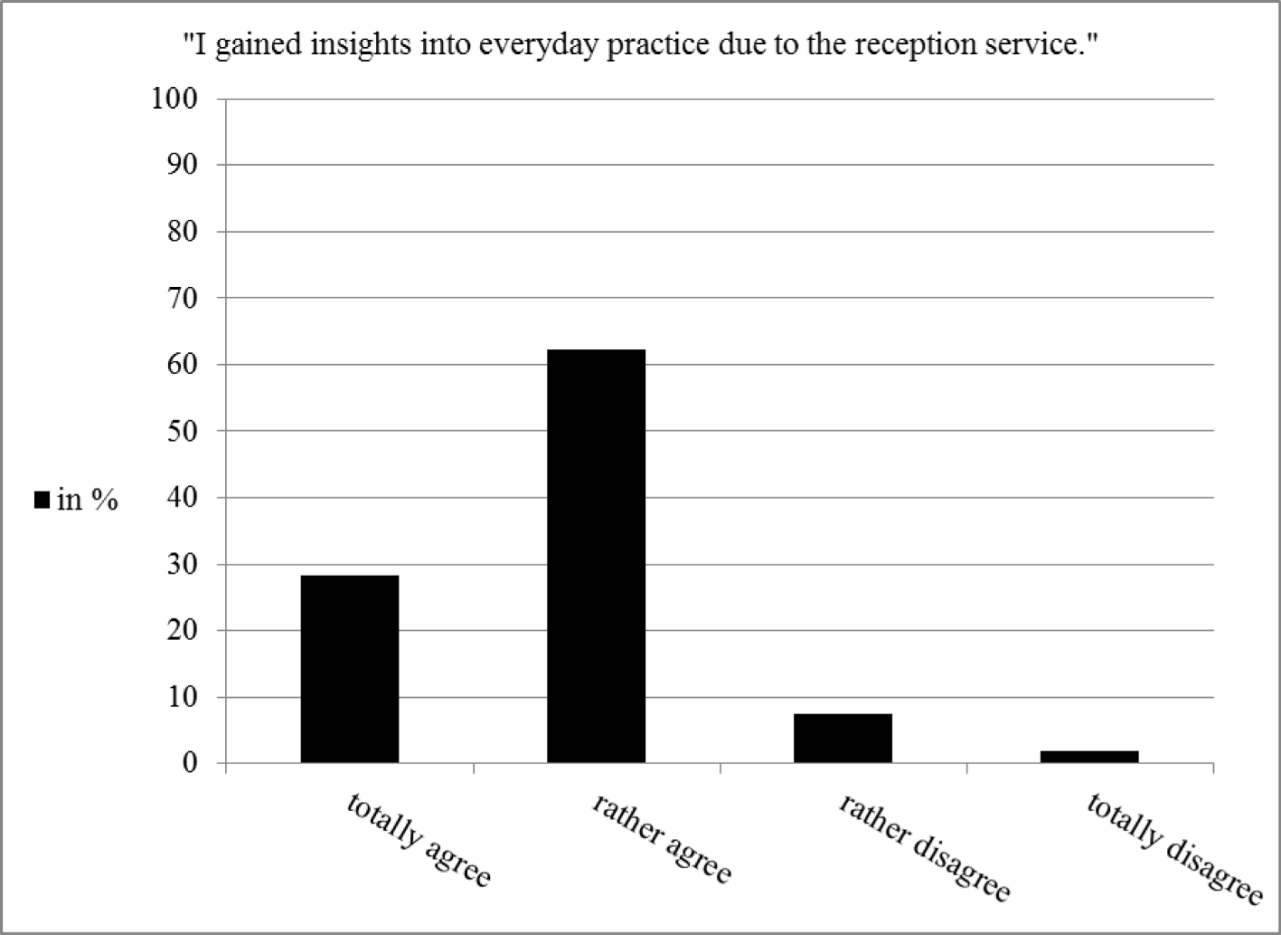
Students‘ assessment concerning insight into everyday practice (n=53)

All in all students reported that they gained insight into everyday practice by participating in the reception service. This is evident from the answers in the questionnaire (see figure 3 (Fig. 3)) and from comments during the interviews. Predominantly mentioned is the scheduling of the patients since the students conducting the reception service did not only have to schedule their own patients but all patients who had an appointment that day and the students had to be available for any questions by the patients.

#### 3.3 Communicative and social competences

**Figure 4:**
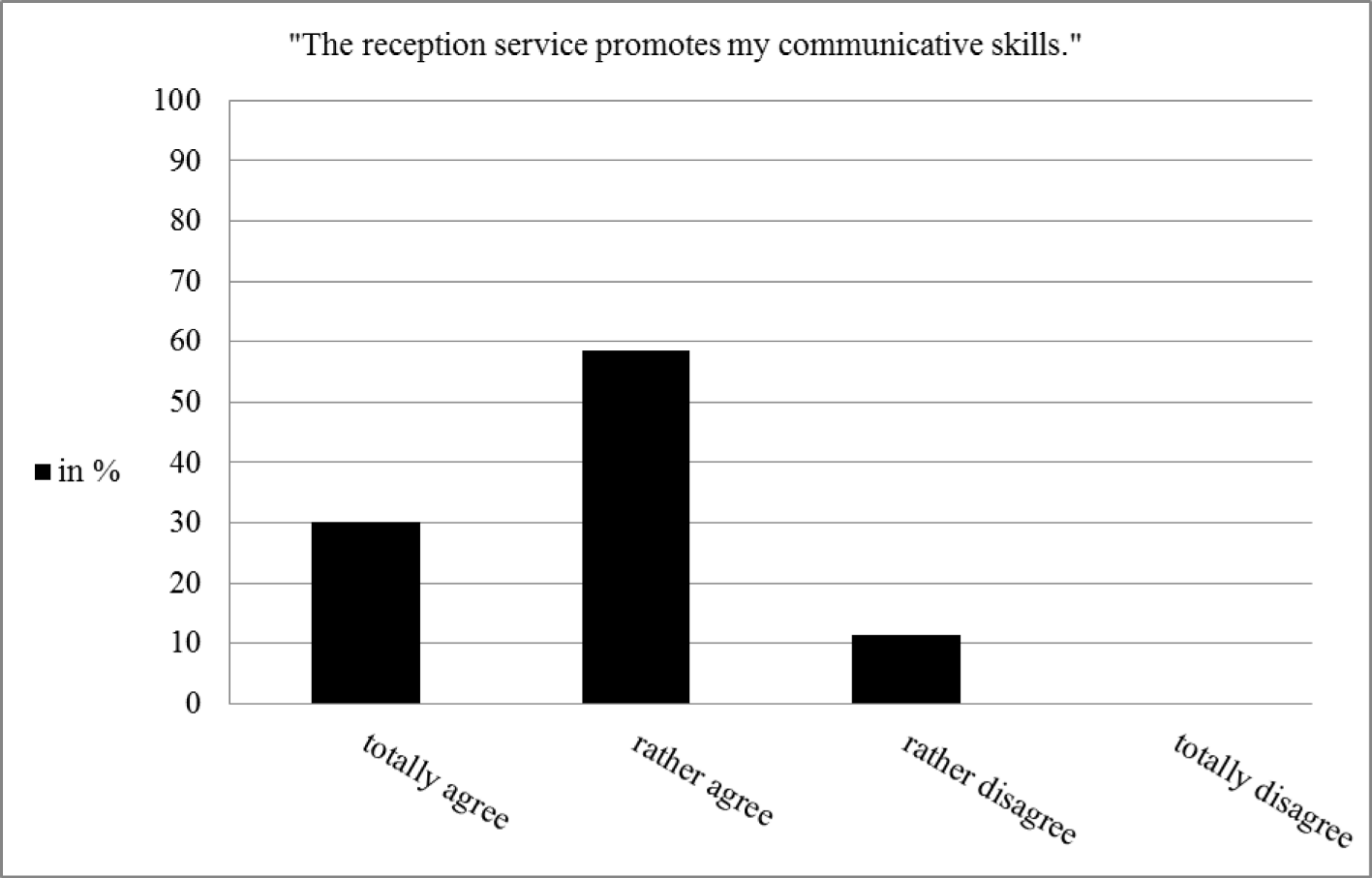
Students‘ assessment concerning the imparting of communicative competences (n=53)

The majority of the students agree that the receptions service promoted their communicative skills (see figure 4 (Fig. 4)). The answers of the four students interviewed provide a first hint from the perspective of students as to which specific competences and in which way they are practised during the reception service. The interviewees report that especially the increase of patient contact is functioning as a training situation regarding social and communicative competences, because the students are confronted with numerous and different types of patient needs (e.g. complaints, questions concerning different aspects of the appointment and the waiting time) than during the rest of the treatment course, where they are confronted only with the dental and medical aspects of the treatment. Furthermore they stated that they experienced a change of perspective with respect to non-dental members of a dental team during the reception service. Due to dealing with tasks usually carried out by non-dental team members, the students put themselves in the position of the non-dental team members and gain a better understanding of their role in and their relevance for the dental team. The Interviewees report that they might benefit from that experience when they start working as a dentist. One student said:

“When I later have my own dental practice […] and I had not the experience of the reception service, I would not know the range of tasks my employees are performing.”

The answers of the students to the questions addressing the training of teamwork skills didn’t correspond with our expectations. The students considered teamwork as an important aspect of the dental profession, but they said the reception service itself had little to do with actual teamwork. One female student said:

 “ […] being in charge of the reception desk has little to do with teamwork. Of course the reception service is carried out by two students and we share the tasks and refer the patients to the appropriate students […]. But that’s all. Teamwork rather plays a major role in the treatment teams.” 

All interviewees stated that the reception service should be retained as a part of the treatment courses, especially because of the possibility to put oneself in the position of non-dental team members and to gain a better understanding of the role they play in a dental team.

## 4. Discussion

Aim of the study was to present development and implementation of the new reception service as a part of the undergraduate treatment courses of the Department of Operative Dentistry and Periodontology, to examine how the reception service is perceived by students and patients and to which extent the reception service is seen as a learning environment for social and communicative competences by the students.

From our point of view we have been successful in offering students through participation in the reception service a novel learning experience as a part of the treatment courses without adding to their workload. The reception service is an innovative addition to the treatment courses and an example for transforming an already existing reality in a course into a new learning environment for students. 

The new facilities of the treatment courses and the cooperative efforts of all departments involved were the preconditions for developing the reception service. Especially the open-minded and cooperative employees of the Administration Department made a successful implementation possible. Preparing the students (communication trainings of the LSK-Dent, trainings how to deal with complains and the possibility to ask the lecturers during the treatment course if questions occur) was obviously sufficient to ensure patient care beyond the actual dental treatment in the treatment course. 

The reception service is positively perceived by patients and students, which reveals high satisfaction among patients and a high level of acceptance by students. 

Students seem to perceive the reception service as a learning environment, where they gain an insight into everyday practice and where they can practise their social und communicative competences. The interviewees reported that they experienced a change of perspective with the non-dental members of a dental team during the reception service promoting an appreciation of the work and the role of the non-dental team members. Due to the small sample, the students’ answers can only be understood as first hints, indicating that the reception service facilitates the “professional behaviour towards all members of the dental team” [[9], p.195] requested by the ADEE. 

Interestingly, even though according to the students experiencing the reception service in general is relevant for future teamwork, they also state that operating the reception desk during the treatment courses has little to do with teamwork. Although this statement seems counterintuitive at first, it might be explained by the general orientation of the students in the treatment courses: the students are treating their patients in a team consisting of two students during the entire course. The students are fixated on this dyadic team since successful collaboration is responsible for a successful patient treatment and hence for passing the course. Additionally the workflow at the reception could play an important role: some of the tasks the students carry out during the reception service could be easily carried out by one person alone, so that basic aspects of teamwork like task and role sharing are only experienced to some extent. The feeling of depending on each other in a team which is associated with task and role sharing is not as present as in the actual dental treatment of a patient, so the necessity of teamwork during the reception service is only perceived partially. There are also hints in literature indicating that dental and medical students underestimate the relevance of teamwork and the education of one’s own teamwork skills in general, since these skills are rather part of the „hidden curriculum“ than imparted explicably [17]. Keeping this information in mind it seems to be important to us to conduct debriefing sessions with the students after their reception service to reflect on the importance of relevant aspects of inter-professional teamwork, and to remind students to become more sensitive to aspects of inter-professional teamwork. Whether such reflection leads to a more complex learning experience during the reception service, as mentioned in the recommendations for teamwork training [18], has to be addressed in further studies.

The low return rates (38% of the patients, 54% of the students) and the small number of interviews have to be factored in when interpreting the results. A selection bias may have occurred since the students cooperated voluntarily. It should be added that the collected data are self-reports by the patients and students [19], [20]. Nevertheless self-reports represent the first level of learning evaluation (reaction and satisfaction; subjective increase in knowledge and skills) according to Kirkpatrick’s evaluation model [21]. To confirm the suspected sustainable effects of the receptions service on teamwork skills and further social and communicative skills and how these skills can be trained during the reception service further studies are needed: Due to the longitudinal design of the curriculum and the fact that the courses interrelate, measuring the output of a single course is only possible within limits. In order to determine the overall increase of students’ competences as a result of attending the longitudinal curriculum, the future plan is to collect data from final year students, who attended the entire course curriculum. 

## Acknowledgements

The authors thank everyone who contributed to the success of the LSK-Dent: Sabine Bornemann, Dr. med. dent. Sonja Derman, Dr. med. dent. Dirk Duddeck, Dr. rer. medic. Franz-Josef Faber, Dr. med. dent. Isabelle Graf , Dr. med. dent. Julia Neuschulz, Dr. med. dent. Sven Scharf, Claus Schlebusch und der Fachschaft Zahnmedizin der Universität zu Köln 

## Competing interests

The authors declare that they have no competing interests.

## Supplementary Material

Details of the reception service

Answer cards filled out by the patients of the treatment coursces

Questionnaire addressing the reception services filled out by the students of the treatment courses

Interview guide

## Figures and Tables

**Table 1:**
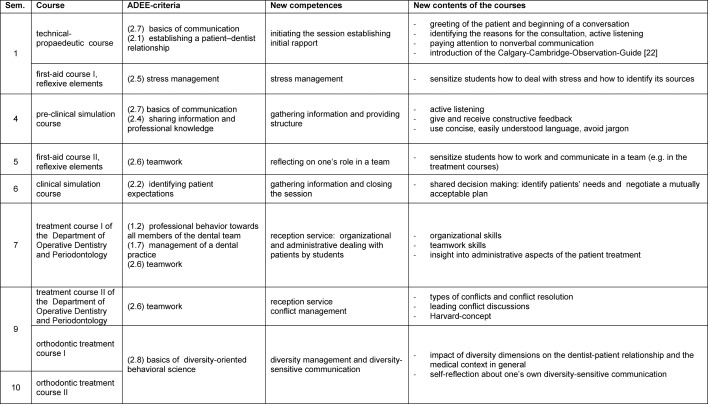
Overview of the course elements of the Longitudinal Curriculum of Social and Communicative Competences for Dentists (LSK-Dent) referring to the respective ADEE-criteria (in brackets: numbers listed in the ADEE-publication [9])

**Table 2:**
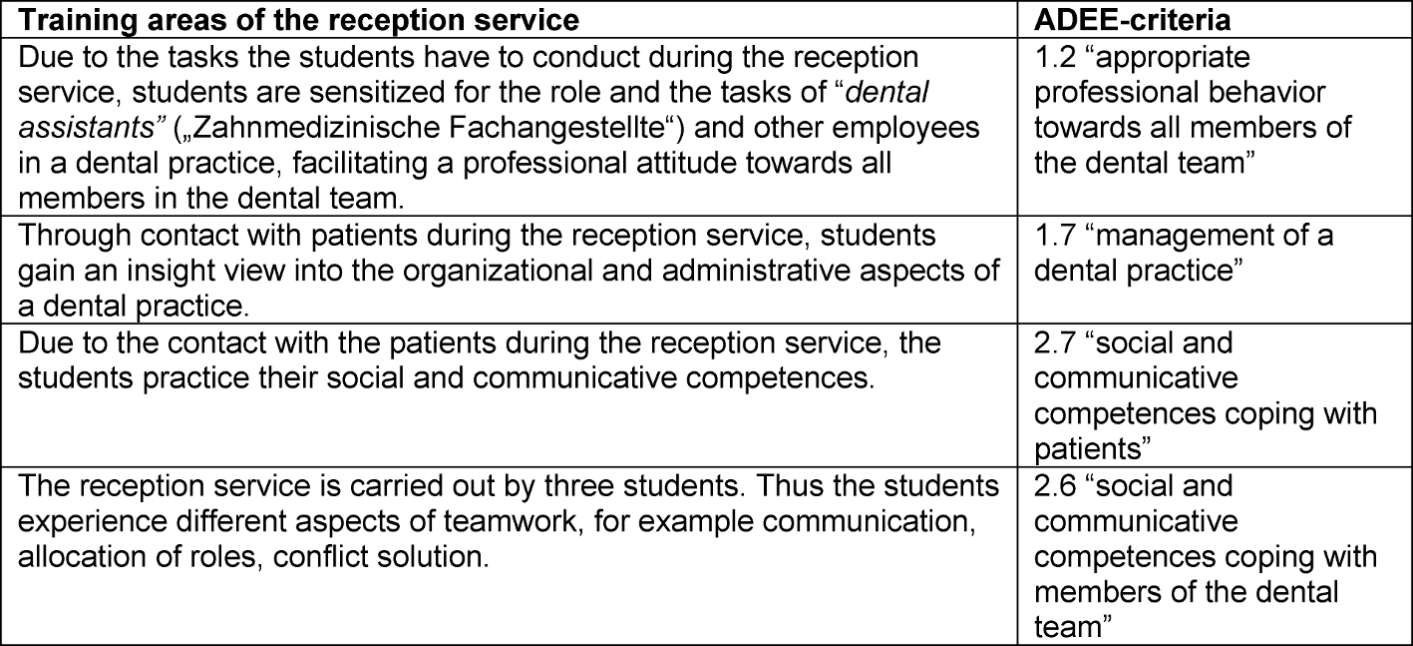
Potential training areas for the reception service, derived from the ADEE-criteria (in brackets: numbers listed in the ADEE-publication [9])
