# Supplementary material for: "May I help you?" – Evaluation of the new student service at the reception desk during the clinical courses at the Department of Operative Dentistry and Periodontology as a part of a longitudinal curriculum of social and communicative competences for dental students
Source: GMS Z Med Ausbild. 2015 Aug 17;32(3):Doc31. doi: 10.3205/zma000973 (PMC4580440; doi:10.3205/zma000973)
Supplement: Details of the reception service [file ZMA-32-31-s-001.pdf]

New registration for patients	To register themselves the patients no longer have to go to the main reception desk in the policlinic anymore and wait there until they get picked up by the students, but can go directly to the new reception desk of the treatment courses and wait in an adjoining waiting room. Using the shared appointment calendar, the students conducting at the reception desk can check with whom the patients have an appointment and inform those students that their patients have arrived.
Swiping health insurance cards	According to employees of the Administration Department swiping the health insurance cards of the patients is not a legitimate task for the students, because it is a part of the billing process and should only be conducted by authorized personnel. Therefore students collect the patients‘ health insurance cards and take them to the main registration of the policlinic. After authorized personnel swiped the cards, the students pick them up and hand them to the students, who are treating the patients. After the dental treatment each student hands out the card to his patient.
Computerised aspects	Since summer semester 2014 the students use the computer program “Comuputer-Konkret” to manage the electronic files of the patients and the appointment calendar.
Credits for conducting the reception service	Conducting the reception service is evaluated as a “*exemplary perfomance”* (“exemplarische Leistung”) in the treatment courses. This means students have to participate in the reception service to pass the course, but they don’t receive a specific amount of credit points for conducting it. All students spend the same amount of time with their patients, ensuring that the reception service does not lead to a surplus load of work for the students.
