# Supplementary material for: "May I help you?" – Evaluation of the new student service at the reception desk during the clinical courses at the Department of Operative Dentistry and Periodontology as a part of a longitudinal curriculum of social and communicative competences for dental students
Source: GMS Z Med Ausbild. 2015 Aug 17;32(3):Doc31. doi: 10.3205/zma000973 (PMC4580440; doi:10.3205/zma000973)
Supplement: Answer cards filled out by the patients of the treatment coursces [file ZMA-32-31-s-002.pdf]

Dear Patient,
We would like to ask you to give us feedback concerning your appointment , so we can optimize the quality of our service.
Thank you.

**1. Was it easy to find the reception desk?**	yes ○	no ○
**2. Please estimate how long you had to wait for your appointment**	________________(minutes)	
**3. How would you rate the waiting time?**	appropriate ○	too long ○

**4. Please rate …**	**no** improvement necessary	**minor** improvement necessary	**some** improvement necessary	**major** improvement necessary	no statement
**a) the greeting /registration at the reception**					
**b) the treatment**					
**c) the discharge at the reception**					

**5. Do you have any suggestions for improvements or other comments?**	

	**very satisfied**	**rather** satisfied	**neither** satisfied nor unsatisfied	**rather** unsatisfied	**very** unsatisfied
**6. How satisfied were you with your appointment overall?**					
