# Supplementary material for: "May I help you?" – Evaluation of the new student service at the reception desk during the clinical courses at the Department of Operative Dentistry and Periodontology as a part of a longitudinal curriculum of social and communicative competences for dental students
Source: GMS Z Med Ausbild. 2015 Aug 17;32(3):Doc31. doi: 10.3205/zma000973 (PMC4580440; doi:10.3205/zma000973)
Supplement: Questionnaire addressing the reception services filled out by the students of the treatment courses [file ZMA-32-31-s-003.pdf]

Dear Students,

We are interested to hear what you think of the reception service as a part of the treatment courses. Therefore we appreciate, if you would fill out the following questionnaire.

Course I ☐ II ☐ this is my first ☐ second ☐ time carrying out the reception service

Mark with a cross where applicable:

	totally agree	rather agree	rather disagree	totally disagree
**I think the reception desk facilitates the patients' registration.**				
**I gained insights into everyday practice due to the reception service.**				
**The reception service promotes my teamwork skills and stress management skills.**				
**The reception service promotes my communicative skills.**				

Do you have any suggestions or further comments?
