# Supplementary material for: "May I help you?" – Evaluation of the new student service at the reception desk during the clinical courses at the Department of Operative Dentistry and Periodontology as a part of a longitudinal curriculum of social and communicative competences for dental students
Source: GMS Z Med Ausbild. 2015 Aug 17;32(3):Doc31. doi: 10.3205/zma000973 (PMC4580440; doi:10.3205/zma000973)
Supplement: Interview guide [file ZMA-32-31-s-004.pdf]

1. Personal details		
**Age**	**Sex**	**Number of reception services:**<br>**Course I:** **Course II:**
**Previous work experience:**<br><br>**Nationality:**<br>**Number of semesters:**		
**2. Overall learning goals**		
• **What did your learn carrying out the reception service?**		
**3. Insight view into everyday practice**		
• **To what extent did you gain an insight view into everyday practice by conducting the reception service?**		
**4. Attitude towards co-workers**		
• **Which relevance do non-dental members of the dental team have?**<br>• **In what way did your experiences from the reception service influence your attitude towards the non-dental members of a dental team?**<br>• **What is the additional benefit of a professional employee in charge of the reception desk for a dental office?**<br>• **Did your appreciation for the non-dental co-workers increase?**		
**5. Teamwork skills**		
**Inter-professional teamwork is a central aspect in a dental office.**<br>• **What do you think are the challenges in a dental team?**<br>• **Which experiences concerning teamwork did you make when conducting the reception service?**<br>• **To what extent did you learn anything about teamwork due to the reception service?**		
**6. Feedback addressing the reception service**		
• **Do you think the reception service should be retained as a part of the treatment courses? Why? Why not?**<br>• **Do you have any suggestions for improvement?**		
